# Effective connectivity underlying neural and behavioral components of prism adaptation

**DOI:** 10.3389/fpsyg.2022.915260

**Published:** 2022-09-02

**Authors:** Selene Schintu, Stephen J. Gotts, Michael Freedberg, Sarah Shomstein, Eric M. Wassermann

**Affiliations:** ^1^Behavioral Neurology Unit, National Institute of Neurological Disorders and Stroke, Bethesda, MD, United States; ^2^Department of Psychological and Brain Sciences, The George Washington University, Washington, DC, United States; ^3^Center for Mind/Brain Sciences-CIMeC, University of Trento, Rovereto, Trentino, Italy; ^4^Laboratory of Brain and Cognition, National Institute of Mental Health, Bethesda, MD, United States; ^5^Department of Kinesiology and Health Education, University of Texas at Austin, Austin, TX, United States

**Keywords:** visuospatial attention, visuomotor adaptation, parahippocampal gyrus, sensorimotor adaptation, posterior parietal cortex, navigation network, cerebellum

## Abstract

Prism adaptation (PA) is a form of visuomotor training that produces both sensorimotor and cognitive aftereffects depending on the direction of the visual displacement. Recently, a neural framework explaining both types of PA-induced aftereffects has been proposed, but direct evidence for it is lacking. We employed Structural Equation Modeling (SEM), a form of effective connectivity analysis, to establish directionality among connected nodes of the brain network thought to subserve PA. The findings reveal two distinct network branches: (1) a loop involving connections from the parietal cortices to the right parahippocampal gyrus, and (2) a branch linking the lateral premotor cortex to the parahippocampal gyrus *via* the cerebellum. Like the sensorimotor aftereffects, the first branch exhibited qualitatively different modulations for left versus right PA, and critically, changes in these connections were correlated with the magnitude of the sensorimotor aftereffects. Like the cognitive aftereffects, changes in the second branch were qualitatively similar for left and right PA, with greater change for left PA and a trend correlation with cognitive aftereffects. These results provide direct evidence that PA is supported by two functionally distinct subnetworks, a parietal–temporal network responsible for sensorimotor aftereffects and a fronto-cerebellar network responsible for cognitive aftereffects.

## Introduction

Prism adaptation (PA) is a classic technique to reversibly modify sensorimotor mapping (Helmholtz, 1867). By practicing pointing movements to visual targets that are laterally shifted, PA modifies sensorimotor coordinates and also alters higher-level cognition in both healthy individuals (Frassinetti et al., 2009; Bultitude and Woods, 2010; Schintu et al., 2018) and patients with hemispatial neglect (McIntosh et al., 2002; Bultitude and Woods, 2010; Jacquin-Courtois et al., 2010). Adaptation to right-shifting prisms (right PA) reduces the pathological rightward bias exhibited by neglect patients (Rossetti et al., 1998), and has become a promising tool for improving negelct symptoms (Luaute et al., 2006; Newport and Schenk, 2012). Adaptation to left-shifting prisms (left PA), by reducing the inherent leftward bias exhibited by healthy individuals called pseudoneglect (Jewell and McCourt, 2000), it causes a visuospatial bias, similar in direction but not in magnitude, to the pathological neglect’s one (Colent et al., 2000; Schintu et al., 2014, 2017; Michel, 2016) and has been extensively employed to model neglect like cognition.

The mechanism of PA has been widely investigated for the last 20 years. Neuroimaging studies have identified the parietal cortex and the cerebellum as key regions (Danckert et al., 2008; Chapman et al., 2010; Küper et al., 2014) but also showed that PA modulates fronto-parietal connectivity (Saj et al., 2013; Schintu et al., 2016; Gudmundsson et al., 2020) and, more generally, that it affects parietal, temporal, and frontal regions associated with spatial attention and awareness (Crottaz-Herbette et al., 2014, 2017; Tsujimoto et al., 2018; Schintu et al., 2020b) and that it even enhances the decoupling between networks such as the default mode and the attention networks (Wilf et al., 2019). However, *how* sensorimotor adaptation to laterally displaced vision leads to cognitive spatial changes is still unclear.

Recently, Panico et al. (2020), based on published neuroimaging and non-invasive brain stimulation studies, put forward a possible framework that would account for the two types of prism-induced aftereffects: the sensorimotor aftereffect usually quantified *via* pointing movements toward a target (Rossetti et al., 1998) and cognitive, aftereffects measured *via* cognitive tasks, such as midline judgment (Colent et al., 2000; Schintu et al., 2014). They proposed that the sensorimotor aftereffect relies on a cerebello-parietal network, whereas the cognitive effects are mediated by bottom-up activation of prefrontal and temporal regions, and that their consolidation involves the motor cortex. However, direct evidence of this hypothesis is lacking.

With the aim of defining the networks underlying PA-induced aftereffects, we used structural equation modeling (SEM; McIntosh and Gonzalez-Lima, 1994; Price et al., 2009; Chen et al., 2011; Reid et al., 2019), a form of effective connectivity analysis (Friston, 1994, 2011; Smith et al., 2012) to uncover connectivity directionality and causality among a set of nodes. We applied SEM on a network we identified in a previous study where we investigated changes in functional connectivity following right and left PA in healthy individuals (Schintu et al., 2020b). In this study, we used a seed-based analysis and found differences in resting-state functional connectivity between regions connected to the posterior parietal cortices (PPC): left cerebellar declive, right parahippocampal gyrus (PHG), and frontal areas such as the right lateral premotor cortex (PMC). While this study revealed differences in resting state functional connectivity between the two groups of healthy individuals adapted to left or right prisms, this type of analysis can only establish patterns of statistical covariation among a set of regions. Effective connectivity, in contrast, attempts to establish networks of causal, directional influences among a set of brain regions. Very little can be said about the nature of the underlying network interactions at the level of functional connectivity alone, as underlying sources/causes can be blurred across multiple regions; to understand the network interactions at a deeper level, effective connectivity estimation is required (e.g., Friston, 2011; Reid et al., 2019).

Here, we set out to model effective connectivity between areas identified in the contrast between left and right PA, along with the PPC seeds for that whole-brain analysis. Based on the recent framework proposed by Panico et al. (2020), we expected to identify separate networks or branches of the model associated with the sensorimotor and cognitive aftereffects.

## Materials and methods

### Participants

Forty right-handed (Edinburgh Inventory; Oldfield, 1971) and right-eye dominant (hole-in-card test; Miles, 1930) healthy adults participated in the original study (Schintu et al., 2020b), approved by the National Institutes of Health, Central Nervous System Institutional Review Board and conducted in accordance with the 1964 Helsinki Declaration (World Medical Association, 2013). Participants were compensated for participation and gave written informed consent. Twenty participants underwent left PA and twenty right PA. After data collection, one participant was excluded because of excessive motion during scans (average motion >0.2 cm) and one because of a congenital cerebral cyst. After effective connectivity analysis criteria were satisfied (see effective connectivity analysis paragraph), the statistical analysis included 36 participants: left PA group (*N* = 18; 11 females; age = 25.9 ± 0.9 SEM) and right PA group (*N* = 18; 12 females; age = 25.7 ± 1.1 SEM). The left and right PA groups did not differ in age (*t*(34) = 0.131, *p* = 0.896). Power calculations for the necessary sample size were derived from a previously published study (Albert et al., 2009) of changes in resting state functional connectivity (pre-to post-training) in a fronto-parietal network with motor learning. The effect size (Cohen’s *d*) of the training-related changes by group in fronto-parietal connectivity was estimated to be 0.9, suggesting that the needed total sample size (two groups combined) to detect an effect of *p* < 0.05 with 80% power was *N* = 39.5 or approximately 20 participants per group.

### Procedures

**Figure 1:**
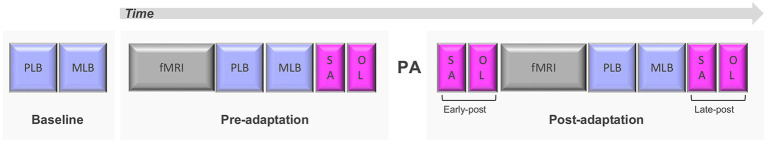
Experimental design. PLB, perceptual line bisection; MLB, manual line bisection; SA, straight-ahead pointing; OL, open-loop pointing; PA, prism adaptation. Adapted from Schintu et al. (2020b).

As reported in Schintu et al. (2020b) the study consisted of two sessions of behavioral testing and functional magnetic resonance imaging (fMRI), one before and one after PA (Figure 1). Prior to each session (baseline) spatial attention was quantified with the perceptual and manual line bisection tasks. In the pre-adaptation session participants underwent the first neuroimaging session, consisting of a resting-state scan (two runs of 5 min) and a population receptive field scan (30 min, reported in Schintu et al., 2022), and the first behavioral assessment consisting of perceptual and manual line bisection tasks, along with two other tasks assessing proprioceptive (straight-ahead pointing task) and visuomotor (open-loop pointing task) performance. Participants were then adapted to left or right-shifting prisms. In the post-adaptation session proprioceptive and visuomotor performance were assessed with the straight-ahead and open-loop pointing tasks immediately after PA (early post-adaptation assessment), then resting-state and population receptive field scans were run, followed by the administration of the perceptual and manual line bisection along with another repeat of the straight-ahead and open-loop pointing tasks (late post-adaptation assessment; Figure 1).

During the behavioral measurement and PA participants seated in front of a horizontal board with their heads supported by a chinrest. See Schintu et al. (2020b) for details.

### Prism adaptation

During PA, participants were fitted with prism goggles deviating the visual field either 15° left or right and performed, with their right hand, 150 pointing movements to the right and left targets, positioned at −10° (left) and + 10° (right) from the participant’s midline. See Schintu et al. (2020b) for details.

### Behavioral assessment

As described in detail in Schintu et al. (2020b), we employed four different tasks quantifying PA-induced aftereffects.

*The perceptual line bisection task,* a modified version of the Landmark task (Milner et al., 1992), was used to measure the visuospatial bias’ perceptual component. Participants judged a series of 66 pre-bisected lines appearing on a computer screen placed in front of them. They were asked to judge whether the mark (transector) was closer to the left or right end of the line. For each participant, the percentage of “right” responses was plotted as a function of the position of the transector. These data were then fitted with a sigmoid function and the value on the x-axis corresponding to the point at which the participant responded “right” 50% of the time was taken as the point of subjective equality (PSE).

*The manual line bisection task* (Schenkenberg et al., 1980) was employed to measure the motor component of the visuospatial bias. Participants judged a series of 10 lines printed on sheets of paper placed on a computer screen in front of them. They were asked to draw a vertical mark where they thought the center of the line was. For each of the ten lines the distance between the mark placed by the participant and the line true center was calculated. The PSE was calculated as the average distance between the true center and the mark draw by the participant, with marks to the right of center coded as positive and to the left as negative.

*The straight-ahead pointing task* was used to measure proprioceptive bias (Rossetti et al., 1998). Participants were asked to point, six times, to their perceived midline with the right index finger at a comfortable and uniform speed while keeping their eyes closed. The proprioceptive bias was measured as the average distance between the landing position and the true midline, with an accuracy of +/− 0.5 cm.

*The open-loop pointing task was* employed to measure the visuomotor bias (similar to Rossetti et al., 1998). Participants were asked to point, six times, to the central target (0 cm) with their right index finger. Vison of the pointing movement and the landing position was occluded. Visuomotor bias was measured as the average distance between the landing position and the central target with an accuracy of +/− 0.5 cm.

### fMRI

Functional and structural MRI data were acquired with a 32-channel head coil on a research-dedicated 3-Tesla Siemens MAGNETOM Prisma MR scanner in the NINDS functional MRI Facility. For each participant, a whole-brain T1-weighted anatomical image (MPRAGE) was obtained, along with a T2* blood oxygen level-dependent (BOLD) resting-state scans. See Schintu et al. (2020b) for details. During resting-state scans, participants were instructed to relax and to look at a cross appearing on a computer screen while thinking about nothing.

#### MRI preprocessing

As reported in Schintu et al. (2020b), functional and structural MRI data were preprocessed using the AFNI software package (Cox, 1996) and following a general preprocessing approach (Wang et al., 2014). The anatomical scans were segmented using Freesurfer (Dale et al., 1999). Two initial volumes were removed from each resting-state run. Volumes were then despiked (3dDespike), slice-time corrected, co-registered to the anatomical scan, transformed to TT_N27 Template space (Talairach and Tournoux, 1988), resampled to 2 mm isotropic voxels, smoothed with an isometric 4-mm full-width half-maximum Gaussian kernel, and scaled to percentage signal change. TRs with head movement >0.3 mm were censored, and simultaneously band-pass filtered (3dTproject) from 0.01 to 0.1 Hz. Six motion parameters and their derivatives were regressed, and were also filtered (0.01 to 0.1 Hz) prior to performing the nuisance regression (e.g., Hallquist et al., 2013; Jo et al., 2013). Measures of mean framewise displacement (@1dDiffMag) and the average voxelwise signal amplitude (standard deviation) were calculated for use as nuisance covariates in group-level analyses (Wang et al., 2014; Gotts et al., 2020).

#### Functional connectivity analysis

The functional connectivity analysis in Schintu et al. (2020b) was a seed-to-whole-brain analysis, with the seeds being the right and left intraparietal sulcus (IPS) regions 1 and 2 combined. These regions of interest (ROI) were chosen because have been shown to affect visuospatial performance when perturbed by TMS (Szczepanski and Kastner, 2013) and were identified using a probabilistic atlas (Wang et al., 2015). The seed-based group analysis identified several additional regions of interest with which they exhibited a significant Phase (pre PA, post PA) x Group (left PA, right PA) interaction. However, since the goal of the current study was to examine effective connectivity models underlying these relationships, we focused the analyses on the largest clusters from the original whole brain analysis reported in Schintu et al. (2020b) that survived both cluster-size correction over a range of voxelwise statistical thresholds and FDR-correction and had a minimum cluster size of 30 voxels (*q* < 0. 005, *p* = 0.00033). The time series data in these ROIs, along with the behavioral data, were those previously reported in Schintu et al. (2020b): the left cerebellar declive, right parahippocampal gyrus (PHG), right lateral premotor cortex (PMC), and the left and right IPS seeds used to detect these regions (the ROI mask used is publicly available on FigShare.com: https://figshare.com/articles/dataset/Frontiers_ROI_mask_N_5_ROIs_/20332218/1). Here, the functional connectivity analysis, unlike the original, examined all possible ROI-ROI connections and provided the experimental data for the effective connectivity modeling. Time series were averaged across voxels for each ROI, and the Pearson’s correlation was calculated among all possible pairs of the five ROIs, followed by the Fisher z-transformation. Only participants satisfying the inclusion criteria for effective connectivity analysis (see effective connectivity analysis paragraph) were included in the functional connectivity analysis. This was conducted using linear mixed-effects models (3dLME), with each pairwise connection’s Fisher z’-transformed Pearson’s correlation serving as the dependent variable, and Group (left PA, right PA), Phase (pre PA, post PA), and their interaction serving as fixed effects, and subjects a random effect. Mean framewise displacement and average voxelwise signal amplitude did not differ between left and right PA groups either pre or post PA (all *p* > 0.14). A false discovery rate (FDR) at *q* < 0.05 was used to correct for multiple comparisons.

#### Effective connectivity analysis

**Figure 2:**
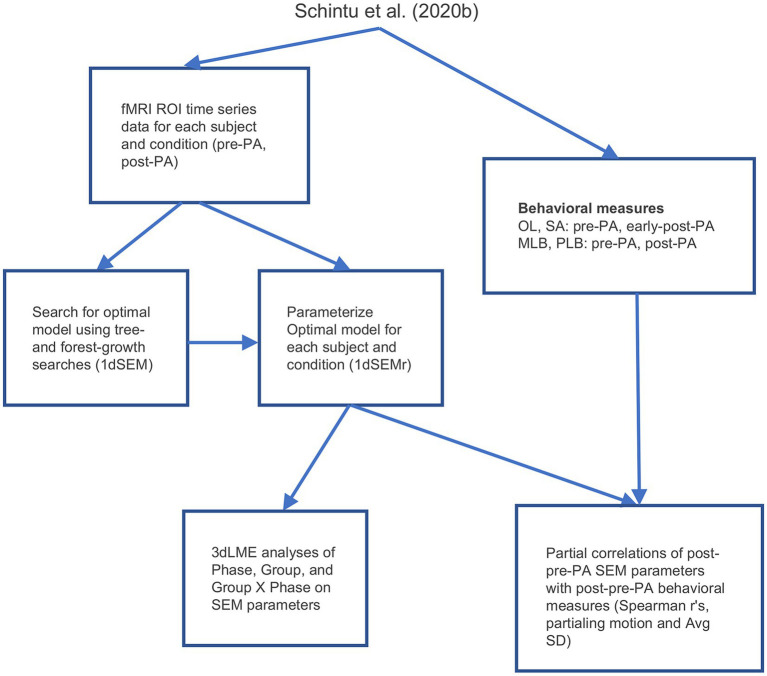
Analysis workflow. PLB, perceptual line bisection; MLB, manual line bisection; SA, straight-ahead pointing; OL, open-loop pointing; PA, prism adaptation; SD, standard deviation; SEM, structural equation modeling.

The five ROIs identified in the whole-brain functional connectivity analyses were submitted to SEM (McIntosh and Gonzalez-Lima, 1994; Price et al., 2009; Chen et al., 2011), a form of effective connectivity analysis (Friston, 1994, 2011; Smith et al., 2012) which detects connectivity directionality and causality. We first performed a search for the model that accounted best for the correlation matrix among the five ROIs when pooling data across all conditions and participants. To do this, the fMRI time series data during rest from the five ROIs were concatenated for each participant for the pre-and post-PA scans, then concatenated further across participants. These data were then submitted to AFNI’s 1dSEM (Chen et al., 2011) to perform an exhaustive search for the optimal SEM model using both tree growth and forest growth algorithms, where optimality was defined as the model that resulted in the smallest out-of-sample generalization error as quantified by the Akaike Information Criterion (AIC; Price et al., 2009), simultaneously maximizing model generalization and minimizing overfitting. The optimal model was then parameterized for each participant’s individual pre and post PA resting-state scans using AFNI’s 1dSEMr (Chen et al., 2011). Only participants with model parameters and post PA versus pre PA contrasts that were less than 3 standard deviations away from the group mean were retained for subsequent analyses, leading to the exclusion of two participants (final sample = 36; see also Gotts et al., 2021). As with functional connectivity, analysis was conducted using linear mixed-effects models (3dLME), with each model parameter serving as the dependent variable, and Group (left PA, right PA), Phase (pre PA, post PA), and their interaction as fixed effects. Partial correlations with behavior were analyzed by calculating the Spearman’s *r* value between the change in model parameters from pre to post PA at each connection (simply post-PA_parameter *minus* pre-PA_parameter) and the change in behavior from pre to post PA (post-PA_behavioral_score *minus* pre-PA_behavioral_score), with mean framewise displacement and average voxelwise signal amplitude removed from the fMRI and behavioral measures as nuisance covariates. For the open-loop and straight-ahead pointing tasks, the early-post-adaptation measures were used as the post PA measure, since this period was the closest in time to the training and maximized the behavioral effects of interest. An FDR at *q* < 0.05 was used to correct for multiple comparisons and two-tailed paired or independent z-tests were carried out for *post hoc* comparisons. An overview of the workflow involved in the effective connectivity analyses is shown in Figure 2.

## Results

### Behavioral measurements

**Figure 3:**
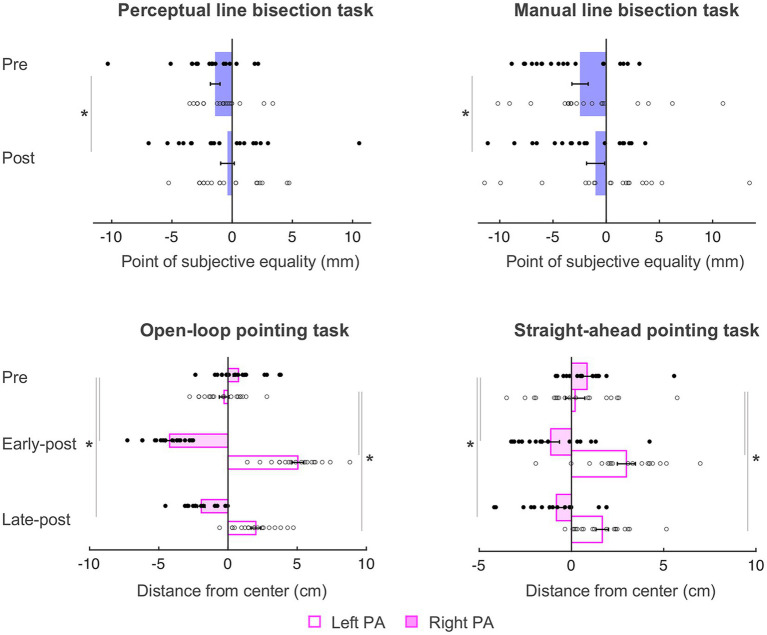
Behavioral results. Negative and positive values represent left and right of the true center (0 cm), respectively. PSE, point of subjective equality; PA, prism adaptation. Error bars represent standard error of the mean. *p < 0.05. Filled circles represent right PA group individual data and open circles represent left PA group individual data. Adapted from figure 2 in Schintu et al. (2020b).

As in Schintu et al. (2020b), the behavioral data were submitted to mixed-model ANOVAs with Phase (pre PA, post PA for perceptual and manual line bisection tasks and pre PA, early-post PA, late-post PA for the straight-ahead and open-loop pointing tasks) as within-subjects variable, and Group (left PA, right PA) as between-subjects variable. The behavioral results summarized here (see Figure 3) are nearly identical to those reported in Schintu et al. (2020b), with the only difference being the total number of participants included (*N* = 36, as opposed to *N* = 38 in Schintu et al., 2020b).

On *the perceptual line bisection task*, there was a main effect of Phase [*F*(1, 34) = 4.712, *p* = 0.037, $\eta_{p}^{2}$ *=* 0.122], such that both groups’ midline judgment shifted rightward of the true center after PA (from − 1.40 to − 0.39 mm), meaning that PA caused a rightward visuospatial bias independently of prism direction. Other main effect or interaction did not reach significance [*F*s ≤ 0.903, *p*s ≥ 0.349].

On *the manual line bisection task*, there was also a main effect of Phase [*F*(1, 34) = 9.105, *p* = 0.005, $\eta_{p}^{2}$ *=* 0.211] such that both groups’ performance shifted rightward of the true center after PA (from − 2.46 to − 0.10 mm). Other main effects or interaction did not reach significance [*F*s ≤ 2.364, *p*s ≥ 0.133].

On *the straight-ahead pointing task*, there was a significant Phase x Group interaction [*F*(2, 68) = 31.320, *p* < 0.001, $\eta_{p}^{2}$ *=* 0.479], such that the left PA group shifted rightward at both early [*t*(17) = −5.722, *p* < 0.001, *Cohen’s d =* 1.35] and late post measurements [*t*(17) = −4.246, *p* = 0.001, *Cohen’s d =* 1.32], and right PA group shifted leftward at both early [*t*(17) = 4.182, *p* = 0.001, *Cohen’s d =* 0.99] and late [−0.81 cm; *t*(17) = 4.880, *p* < 0.001, *Cohen’s d =* 0.96] post measurements, meaning that left and right PA affected proprioceptive performance in the expected direction and that both groups were adapted until the end of the experiment. There was also a main effect of Group [*F*(1, 34) = 15.276, *p* < 0.001, $\eta_{p}^{2}$ *=* 0.310] indicating a general rightward pointing for the left PA group and leftward pointing for the right PA group and no main effect of Phase [*F*(2, 68) = 1.517, *p* = 0.227]. As in Schintu et al. (2020b), the amount of adaptation (i.e., absolute value) did not differ significantly between groups at either time point [*ts* ≤ 0.803, *p*s ≥ 0.428].

On *the open-loop pointing task*, there was a significant Phase x Group interaction [*F*(2, 68) = 278.251, *p* < 0.001, $\eta_{p}^{2}$ *=* 0.891], with the left PA group shifting rightward at the early [*t*(17) = −17.822, *p <* 0.001, *Cohen’s d =* 4.20] and late *t*(17) = −9.054, *p* < 0.001, *Cohen’s d =* 4.11] post measurements, and the right PA group shifting leftward at the early [*t*(17) = 12.196, *p* < 0.001, *Cohen’s d =* 2.87] and late [−1.93 cm; *t*(17) = 9.694, *p* < 0.001, *Cohen’s d =* 2.81] post measurements, meaning that left and right PA affected visuomotor performance in the expected direction and that both groups were adapted until the end of the experiment. There was a main effect of Group [*F*(1,34) = 86.675, *p* < 0.001, $\eta_{p}^{2}$ = 0.718] indicating a general rightward pointing for the left PA group and leftward pointing for the right PA group, and no main effect of Phase [*F*(2, 68) = 1.455, *p* = 0.241]. Again, the amount of visuomotor adaptation (i.e., absolute value) did not differ between groups [*ts* ≤ −1.035, *p*s ≥ 0.308].

### Resting-state functional connectivity

**Figure 4:**
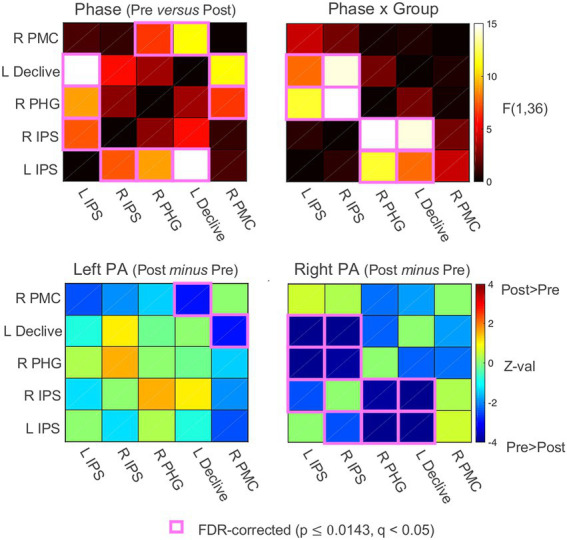
Functional connectivity results. Functional connectivity correlation matrices. PA, prism adaptation. Pre, before PA; Post, after PA; IPS, intraparietal sulcus; PHG, parahippocampal gyrus; PMC, premotor cortex. See also Supplementary material.

To characterize the changes in functional connectivity among the five ROIs that survived the whole-brain linear mixed-effects (LME) regression analysis (Schintu et al., 2020b), Pearson’s correlations among the ROI time series were calculated for each participant, Fisher’s z’ transformed, and then submitted to linear mixed-effects analyses with Group (left PA, right PA), Phase (Pre PA, Post PA), and their interaction as fixed effects. While there were no significant main effects of Group, there were significant main effects of Phase involving the left IPS with the right IPS, the right PHG, and the left declive, and the right lateral PMC with the right PHG and left declive (FDR-corrected to *q* < 0.05; all *p*s ≤ 0.014). These main effects of Phase corresponded to decreases in connectivity from Pre to Post PA irrespective of PA direction (Figure 4). However, as expected from the original seed-based results (Schintu et al., 2020b) there were significant Phase x Group interactions observed for both the left and right IPS ROIs with the right PHG and left declive, and a trend-level interaction involving the left IPS and the right lateral PMC (*p* = 0.044, uncorrected). The significant and corrected interactions appeared to be driven exclusively bya decrease in functional connectivity from Pre to Post PA involving the bilateral IPS and the right PHG and left declive in the right PA group (z-tests, all *p* < 0.001), with non-significant changes at the same connections for the left PA group.

### Resting-state effective connectivity

**Figure 5:**
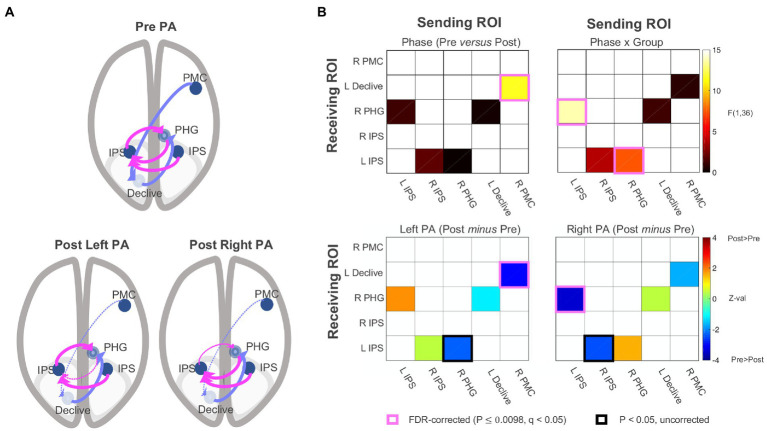
Effective connectivity results. **(A)** Five-parameter model identified by statistical equation modeling (top) and changes post PA relative to pre PA after left and right PA (bottom panels). Dotted line = Decrease in effective connectivity related to the significant main effect of Phase and Phase X Group interactions shown in **(B)**. Effective connectivity correlation matrices. PA, prism adaptation. IPS, intraparietal sulcus; PHG, parahippocampal gyrus; PMC, premotor cortex. See also Supplementary material.

The functional connectivity data were submitted to SEM (McIntosh and Gonzalez-Lima, 1994; Price et al., 2009; Chen et al., 2011; Reid et al., 2019) using AFNI’s 1dSEM and 1dSEMr. The model search process (see effective connectivity analysis paragraph) arrived at a five-parameter model involving the following directional connections: (1) left IPS → right PHG, (2) right IPS → left IPS, (3) right PHG → left IPS, (4) left declive → right PHG, and (5) right lateral PMC → left declive. As shown in Figure 5, the model had two branches interacting through the right PHG, (1) a loop between left IPS and right PHG, with additional input to left IPS from right IPS, and (2) a feedforward branch from right lateral PMC to left declive to right PHG. The model was then parameterized for each participant’s experimental conditions (e.g., left PA, pre PA), and each parameter served as a dependent variable in linear mixed-effects modeling for group analysis, with Phase, Group, and their interaction as fixed effects. No connections in the pre and post PA conditions were found to be significantly negative in the left PA or right PA groups and only excitatory connections were detected. As with functional connectivity, there were no significant main effects of Group. There was a significant main effect of Phase with no Phase x Group interaction (*p* = 0.343) for the right lateral PMC → left declive connection [*F*(1,34) = 11.628, *p* = 0.002, *q* < 0.05], corresponding to a significant decrease in effective connectivity from pre to post adaptation in the left PA group (*p* = 0.002, *q* < 0.05) and a non-significant trend for decreased connectivity in the right PA group (*p* = 0.085). There were significant Phase x Group interactions for left IPS → right PHG [*F*(1,34) = 13.639, *p* < 0.001, *q* < 0.05] and the right PHG → left IPS connections [*F*(1,34) = 7.498, *p* = 0.001, *q* < 0.05]. For left IPS → right PHG, there was a non-significant trend for increased connectivity in the left PA group (*p* = 0.068) and a significant decrease for the right PA group (*p* < 0.001, *q* < 0.05). The reverse of this pattern was observed for the right PHG → left IPS connection, with a decrease in connectivity for the left PA group (*p* = 0.022) and a numerical increase in connectivity for the right PA group (*p* = 0.11). Taken together, the results indicate that, for the left PA group, drive is relatively reduced into the left IPS from the right PHG, whereas for the right PA group, drive is relatively reduced out of the left IPS into the right PHG, having qualitatively opposing changes on the flow of activity within the circuit. The feedforward flow from right lateral PMC to left declive, and, indirectly, the right PHG, decreased after PA independent of direction.

### Brain-behavior correlations

**Figure 6:**
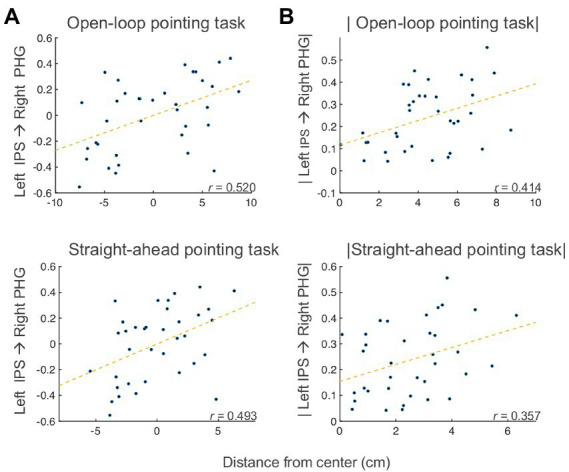
Bain-behavior correlation. **(A)** Partial correlations between post *minus* pre PA changes in left IPS → right PHG connection and open-loop pointing (top) and straight-ahead pointing (bottom) tasks. **(B)** Partial correlations between post *minus* pre PA changes (absolute values) in left IPS → right PHG connection and open-loop pointing (top) and straight-ahead pointing (bottom) tasks. IPS, intraparietal sulcus; PHG, parahippocampal gyrus.

To examine the associations between effective connectivity and behavior, we measured correlations between the changes (post PA *minus* pre PA) in open-loop pointing, straight-ahead pointing, perceptual and manual line bisection tasks, and changes in the SEM model, partialling motion, and average voxelwise standard deviation. We found significant partial correlations between change in left IPS → right PHG connection and the amount the open-loop and straight-ahead pointing measures [open-loop: Spearman’s partial *r*(32) = 0.520 (95% CI: 0.2206, 0.7298), *p* = 0.002, *q* < 0 0.05; straight-ahead: Spearman’s partial *r*(32) = 0.493 (95% CI: 0.1858, 0.7124), *p* = 0.003, *q* < 0.05; Figure 6A]. The positive sign of these correlations indicates that as drive from left IPS to right PHG increased after PA, the behaviors showed a rightward shift across participants, with the reverse following a decrease in drive from left IPS to right PHG. The right IPS → left IPS connection was positively correlated with the pointing measures [open-loop: Spearman’s partial *r*(32) = 0.300 (95% CI: −0.0425, 0.5794), *p* = 0.084; straight-ahead: Spearman’s partial *r*(32) = 0.356 (95% CI: 0.0203, 0.6196), *p* = 0.039], whereas the right PHG → left IPS connection was negatively related [open-loop: Spearman’s partial *r*(32) = −0.317 (95% CI: −0.5917, 0.0237), *p* = 0.067; straight-ahead: Spearman’s partial *r*(32) = −0.380 (95% CI: −0.6364, −0.048), *p* = 0.026]. The FDR-corrected effects involving the left IPS → right PHG connection were further checked to make sure the correlations were not due to the different behavioral center offsets in the left PA and right PA groups by applying the absolute value function to the behavioral and SEM Post PA *minus* Pre PA changes. For both the open-loop and straight-ahead pointing measures, these correlations with left IPS → right PHG remained significant [open-loop: Spearman’s partial *r*(32) = 0.413 (95% CI, 0.087, 0.6591), *p* = 0.015; straight-ahead: Spearman’s partial *r*(32) = 0.356 (95% CI, 0.0203, 0.6196), *p* = 0.039] (see Figure 6B), meaning that that the correlation was not driven by the opposite direction of the changes in both connectivity and behavior. Taken together, these results suggest that the behavioral effects of PA on open-loop and straight-ahead pointing are mediated by drive from the left IPS to the right PHG, with greater drive leading to a rightward shift, in a manner qualitatively similar to the group-level effects.

While no correlations with the perceptual and manual line bisection measures survived correction, one non-significant trend was observed between the perceptual line bisection post PA *minus* pre PA change and the right lateral PMC → left declive connection [Spearman’s partial *r*(32) = 0.318 (95% CI: −0.0226, 0.5925), *p* = 0.066], suggesting the involvement of the second branch in the cognitive aftereffect.

## Discussion

The aim of this study was to investigate the network underlying the sensorimotor and cognitive aftereffects of PA. We employed SEM to estimate connectivity directionality in the set of clusters that survived the left PA versus right PA contrast in the whole brain functional connectivity analysis (Schintu et al., 2020b). Based on a recently proposed framework (Panico et al., 2020), we hypothesized that these effects rely on two distinct parts of the network.

At the behavioral level, the group adapted to left prisms showed a significant rightward sensorimotor aftereffect, whereas the group adapted to right prisms exhibited a significant leftward sensorimotor aftereffect, as measured by open-loop and straight-ahead pointing tasks (Schintu et al., 2020b). Both groups of participants were significantly adapted until the end of the experiment and the amount of adaptation did not differ between the two PA directions, ensuring that any possible difference at the neural level would not be due to a different level of sensorimotor adaption achieved by the two groups. Concerning the cognitive aftereffect, while we expected a rightward bias shift on the perceptual line bisection (but not manual line bisection McIntosh et al., 2019; Schintu et al., 2020a), following left, but not right, PA (Colent et al., 2000; Schintu et al., 2014, 2017, 2018), both groups judged the center of a line significantly more rightward than they did at baseline. The lack of a significant rightward shift following left PA could be due to fluctuations of the cognitive aftereffect (Schintu et al., 2014) and does not invalidate the effects on functional connectivity, since also others (see Crottaz-Herbette et al., 2014) have reported significant effects of PA on brain activity in the absence of significant behavioral changes.

At the neural level, the functional connectivity analysis examining all possible connections among the five ROIs replicated the original finding that the Phase x Group interaction was associated with changes involving bilateral IPS, right PHG, and left declive after right PA (Figure 4). The effective connectivity analysis revealed that the optimal solution was a five-parameter model with two separate branches interacting through the right PHG: (1) a loop between left IPS and right PHG, with additional input to left IPS from right IPS, and (2) a feedforward branch from right lateral PMC to left declive to right PHG (Figure 5).

Concerning the first branch, results indicate that the drive from left IPS to right PHG decreased following right PA, and the drive decreased in the opposite direction for left PA (right PHG → left IPS). Importantly, the amount of change in effective connectivity for the left IPS to right PHG connection correlated with measures of sensorimotor aftereffects (Figure 6). The positive sign of the correlations indicate that, as the drive from left IPS to right PHG increases(/decreases) after PA, the behaviors exhibit a rightward (/leftward) shift. The characterization of the first branch as the neural basis of the sensorimotor aftereffect is further supported by the significant correlations between the sensorimotor aftereffect, as indexed by the straight-ahead pointing performance, and the connection from right to left IPS and from right PHG to left IPS. It is not surprising that these other two connections show similar behavioral correlations with straight-ahead pointing performance, although at lower magnitudes, given the similar model function of the connections forming a loop among the left and right IPS and the right PHG (changing drive between the IPS and the PHG; Figure 6).

Regarding the second branch, we found significantly decreased connectivity following PA which, like the cognitive aftereffect, was independent of PA direction. Consistent with the literature mainly showing no effects of right PA on spatial bias in healthy individuals (Colent et al., 2000; Bultitude and Woods, 2010; Schintu et al., 2014, 2017), the exploratory *post-hoc* comparison revealed that the general decrease in effective connectivity from right PMC to the left declive was driven by the left PA group. Furthermore, this feedforward connection was marginally correlated with the amount of change in midline judgment measured by perceptual line bisection. The main effect of Phase on effective connectivity in the second branch of the network parallels the cognitive aftereffects. While no significant cognitive aftereffect for the right PA group was expected, we did expect a significant change in visuospatial bias after left PA (Schintu et al., 2014, 2017, 2020b). The absence of a visuospatial effect for the left PA group strong enough to drive a Phase x Group interaction could be due to the >35 min elapsed from adaptation, since it has been shown that the PA-induced cognitive effect lasts around 35 min (and less than 1 h) and fluctuates (Schintu et al., 2014, 2017). Thus, while tentative, the possible involvement of the second branch in the cognitive effect of prism adaptation suggests a possible division of labor between the two branches of the model, with the involvement of the IPS to PHG loop in the sensorimotor aftereffect and the right PMC to PHG branch in the cognitive aftereffect.

The involvement of the cerebellum in PA is well recognized (Weiner et al., 1983; Pisella et al., 2004). However, whether it is involved in the early *recalibration phase*, which allows strategic correction of pointing movement, or in the later *realignment phase*, which consists of automatic remapping and thought to be the basis of the cognitive aftereffect (Redding et al., 2005; Redding and Wallace, 2006), or in both phases (Panico et al., 2016), has been debated (Luauté et al., 2009; Chapman et al., 2010; Küper et al., 2014). One study (Panico et al., 2016) found cerebellar involvement in both phases, however, it only quantified the sensorimotor aftereffect. The cerebellum is involved in the second branch of the model, which is the one we propose to be responsible of the emergence of the cognitive aftereffect, in agreement with the emerging recognition of cerebellar involvement in cognition (Rondi-Reig and Burguière, 2005).

Earlier models have suggested that the mechanism of PA relies on inhibitory, bottom-up, signaling from the cerebellum to the PPC (Pisella et al., 2006; Striemer and Danckert, 2010). All the connections identified in this study are positive, meaning that none of the connections are effectively inhibitory. Instead of a direct connection between the cerebellum and PPC, the effective connectivity modeling identified one between the cerebellum and the parahippocampal gyrus. The PPC is crucial in generating PA aftereffect and the recurrent connections involving the left and right IPS present in our findings further support its central role. Surprisingly, however, the present findings also suggest that the PPC is not the region shared between the two model branches, but that it is only involved in the first branch and thus only in the sensorimotor aftereffect. The hub of the model appears to be the PHG which, along with the PPC, is involved in spatial navigation (Aguirre et al., 1996; Aguirre and D’Esposito, 1997; Maguire et al., 1998) and allocentric (world-referenced) representation (Aguirre and D’Esposito, 1999). Clearly, its role in both sensorimotor and cognitive PA-induced aftereffect requires further investigation and confirmation.

It is important to consider that effective connectivity does not necessarily correspond to physical connectivity, but rather to causal physiological connections. Therefore, there might be connections or ROIs that are not included in the present modeling. SEM is a statistical method for evaluating causality in connectivity and while it provides a window into the mechanism of action of PA, it requires confirmation with anatomical and/or physiological methods. Finally, despite right PA having become the gold standard control condition for left PA, it has consistently failed to produce significant cognitive changes in healthy individuals (Colent et al., 2000; Michel et al., 2003; Loftus et al., 2009; Bultitude and Woods, 2010; Reed and Dassonville, 2014; Schintu et al., 2014, 2017, 2018), not having compared the left PA to a neutral (no shift) group might have prevented the identification of the plastic mechanisms common to both PA directions.

## Conclusion

In conclusion, this is the first study that investigates the effective connectivity underlying PA and identifies separate subnetworks for the sensorimotor and cognitive aftereffects. These novel findings call for refinement of the current model of PA and further investigation of roles of the PHG and cerebellum in PA and in visuospatial attention more generally.

## Data availability statement

The raw data supporting the conclusions of this article will be made available by the authors, without undue reservation.

## Ethics statement

The studies involving human participants were reviewed and approved by the National Institutes of Health, Central Nervous System Institutional Review Board. The participants provided their written informed consent to participate in this study.

## Author contributions

SSc, EW, and SSh contributed to the conception and design of the study. SSc collected the data. SSc, SG, and MF performed imaging and statistical analysis. SSc and SG wrote the first draft of the manuscript. All authors contributed to the article and approved the submitted version.

## Funding

This work was supported by the National Science Foundation (grant number BCS-1921415 to SSh and BCS-2022572 to SSh), the National Institutes of Health Ruth L. Kirschstein National Research Service Award (to SSc), the Clinical Neurosciences Program of the National Institute of Neurological Disorders and Stroke (to EW), and the NIMH Intramural Research Program (to SG).

## Conflict of interest

The authors declare that the research was conducted in the absence of any commercial or financial relationships that could be construed as a potential conflict of interest.

## Publisher’s note

All claims expressed in this article are solely those of the authors and do not necessarily represent those of their affiliated organizations, or those of the publisher, the editors and the reviewers. Any product that may be evaluated in this article, or claim that may be made by its manufacturer, is not guaranteed or endorsed by the publisher.
